# Reply to T. Hasegawa et al and I. Gross et al

**DOI:** 10.1200/JCO.2016.68.7681

**Published:** 2016-08-08

**Authors:** Brian Leyland-Jones, Els Vercammen, Liang Xiu

**Affiliations:** Brian Leyland-Jones, Avera Cancer Institute, Sioux Falls, SD; Els Vercammen and Liang Xiu, Janssen Research & Development, Raritan, NJ

We have several responses to the correspondence by Gross et al^1^ on the critical role of iron in our study.^2^ In contrast to what the authors stated, iron parameters at baseline and iron use during the study were provided in the Data Supplement,^2^ and use of iron during the study is adequately described in the protocol (NT00338286 and EudraCT Number 005-001817-17). Patients in both treatment arms with transferrin < 20% were to be considered to have functional iron deficiency and were to receive iron therapy, preferably by intravenous administration. The recommended iron therapy was in agreement with the European guidelines for treatment of renal anemia at the start of the study^3^ and aligned with the European Organisation for Research and Treatment of Cancer guidelines on iron supplementation in patients with cancer who received chemotherapy and erythropoiesis-stimulating agents (ESAs).^4^

The recommendation of iron use in this study is the same as in another metastatic breast cancer study with ESAs, the Breast Cancer-Anemia and the Value of Erythropoietin study.^5^ This study reported concomitant iron supplementation of 85% in the epoetin beta group and 80% in the control group. Of note, in the Breast Cancer Erythropoietin Survival Trial (BEST) study,^6^ a daily dose of elemental iron (oral) 200 mg was recommended when transferrin was < 20%; only 1.8% of the total population received iron supplementation.

Iron therapy in patients with cancer has often been contentious because of its potential contribution to tumor initiation and tumor growth, its role in the tumor microenvironment and metastasis, and the changes in the uptake and management of iron in patients with cancer.^7^ Particularly in breast cancer, almost 50% of all genes that are involved in the regulation or maintenance of iron metabolism were significantly associated with clinical outcome.^8^ We believe these findings may contribute to the cautious use of iron supplementation in patients with cancer.

The comment by Gross et al^1^ regarding thrombotic vascular events and target hemoglobin (Hb) is interesting. As RBC transfusion guidelines with respect to initiation and/or maintenance Hb are quite different from the guidelines for anemia management with ESAs in patients with cancer, the Hb reached in the best standard care (BSC) group is obviously lower compared with the epoetin alfa (EPO) group. Whether this may potentially bias the reporting of thrombotic vascular events in both groups remains hypothetical.

Hasegawa et al^9^ provide an alternative approach to analyzing and interpreting the data presented in our study.^2^ The study was designed to show noninferiority of EPO to BSC in terms of progression-free survival (PFS) in patients with metastatic breast cancer. The prespecified noninferiority margin, as hazard ratio (HR; EPO *v* BSC), was 1.15. The final analysis of the study was based on a total of 2,098 patients and 1,659 PFS events. Median PFS was 7.4 months in both treatment groups and HR was 1.089 with a 95% CI of 0.988 to 1.200.

By using restricted mean survival time (RMST) approach, Hasegawa et al^9^ showed that, with up to 48 months of follow-up, the EPO group had an average PFS of 9.9 months compared with 11.4 months in the BSC group. The difference of the two averages was 1.5 months, with a 95% CI of 0.5 to 2.6. This analysis was based on individual-level data that were reconstructed from the published results.

We have conducted a similar post hoc analysis on the basis of original patient-level data up to a follow-up time of 75 months. Our results showed that the EPO group had an average PFS of 10.1 months compared with 11.6 months in the BSC group. The difference of the two averages was 1.6 months, with a 95% CI of 0.45 to 2.66. Our analysis and that of Hasegawa et al^9^ have closely matched results, although conducted independently with different data sources and different follow-up times.

On the basis of HR, there was an observed 9% increase in risk of progression or death for the EPO group compared with BSC, and the study did not achieve the noninferiority objective per the prespecified noninferiority margin of 1.15. As Hasegawa et al^9^ rightly pointed out, these are not probability assessments and they lack intuitive clinical interpretations. Results based on the RMST analysis by Hasegawa et al^9^ showed an observed absolute 1.5 months, or 13% (1.5 divided by 11.4), relative decrease in average time of PFS for EPO compared with BSC. The study noninferiority objective cannot be evaluated on the basis of this result as there was no prespecified noninferiority margin for analysis with RMST, but the outcome has statistically ruled out a decrease in average time of PFS > 2.6 months or < 0.5 months.

HR, under specific assumptions in the underlying distribution, can be considered the inverse of the median time ratio of the two pertinent treatment groups. However, these assumptions are more often than not unsatisfied, and the median as a single cross-sectional measure by no means reflects the entire time-to-event trajectory.

The most notable advantages of the RSMT approach are that the validity of the method is independent of any assumptions regarding the underlying distribution and that the clinical interpretation of the results is easy and intuitive. Determining the follow-up cutoff requires careful planning at the study design stage as it could introduce bias in favor of or against one treatment arm versus the other. For a noninferiority study, determination and justification for the noninferiority margin in terms of RMST may not be an easier matter even though the method has a more intuitive appeal.

With regard to identification of a subgroup of patients that would not have the safety concerns,^9^ our study^2^ included analyses for eight prespecified subgroups defined by demographic and baseline characteristics (age, body mass index, Eastern Cooperative Oncology Group, line of chemotherapy, human epidermal growth factor receptor 2 [HER2]/neu status, time from initial diagnosis to metastatic disease, type of prior adjuvant therapy, and Hb level) and three additional subgroups based on hormone receptors and HER2 status (HER2 positive, hormone receptor positive or HER2 negative, and triple negative). Results from these subgroup analyses were consistent, in general, with that of the overall population.
